# Immortelle (*Helichrysum italicum* (Roth) G. Don) Essential Oil Showed Antibacterial and Biofilm Inhibitory Activity against Respiratory Tract Pathogens

**DOI:** 10.3390/molecules27175518

**Published:** 2022-08-27

**Authors:** Viktória L. Balázs, Rita Filep, Fanni Répás, Erika Kerekes, Péter Szabó, Béla Kocsis, Andrea Böszörményi, Judit Krisch, Györgyi Horváth

**Affiliations:** 1 Department of Pharmacognosy, Faculty of Pharmacy, University of Pécs, 7624 Pécs, Hungary; 2 Department of Microbiology, Faculty of Science and Informatics, University of Szeged, 6726 Szeged, Hungary; 3 Institute of Geography and Earth Sciences, Faculty of Sciences, University of Pécs, 7624 Pécs, Hungary; 4 Department of Medical Microbiology and Immunology, Medical School, University of Pécs, 7624 Pécs, Hungary; 5 Institute of Pharmacognosy, Faculty of Pharmacy, Semmelweis University, 1085 Budapest, Hungary; 6 Department of Food Engineering, Faculty of Engineering, University of Szeged, 6724 Szeged, Hungary

**Keywords:** essential oil, respiratory tract, immortelle, antibacterial activity, biofilm inhibition

## Abstract

The biofilm formation of bacteria in different parts of the human body can influence the success of antibiotic therapy. Essential oils (EOs) and their components are becoming increasingly popular in point of view of medicinal applications, because of their antibacterial efficacy. The immortelle EO has been used traditionally as an expectorant; however, there are no studies summarizing its antibacterial effect against respiratory tract bacteria. Our aim was to investigate the antibacterial and biofilm inhibitory activity of immortelle (*Helichrysum italicum*) EO against respiratory tract pathogens such as *Haemophilus influenzae*, *H. parainfluenzae*, *Pseudomonas aeruginosa* and *Streptococcus pneumoniae*. In order to prove the antibacterial effect of the immortelle EO, broth microdilution and biofilm inhibition tests, and membrane damage assay were investigated. Scanning electron microscopy was used to identify the structural modifications in bacterial cells. Our results showed that immortelle EO has antibacterial and anti-biofilm effects against respiratory tract bacteria used in this study. *H. parainfluenzae* was the most sensitive to each treatment, however, *P. aeruginosa* was the most resistant bacteria. In conclusion, the studied EO may have a role in the treatment of respiratory tract infections due to their antibacterial and anti-biofilm activity.

## 1. Introduction

Essential oils (EOs) are odorous liquids with multi-component composition and produced from different plant parts [1]. Due to their natural origin and disinfectant property, EOs have become more and more widespread in the preservation of food products, and the prevention of various diseases [2]. It has already been proved that certain EOs also have antibacterial, antifungal, and antiviral effects [3].

Recently, antibacterial resistance become a major problem [4,5], which is in correlation with the biofilm-forming ability of bacteria. The background of most chronic infections is that the bacteria are much more resistant to adverse environmental effects when organized into a biofilm, so disinfectants and antibiotics are not suitable to suppress them. The bacterial biofilm is a community of bacterial cells surrounded by a polymer matrix produced by them [6]. Due to the continuous growth and ripening, the thickness of the biofilm can reach 100 µm. The bacteria that make up a biofilm begin their coordinated action in terms of metabolism, growth, and defense. One of the most fundamental and important structural features of biofilms is that the bacterial cells are quite close to each other. This, in itself, promotes survival, as the process of horizontal gene transfer can take place quickly and easily [7].

The respiratory tract is the barrier between the environment and the human body, which may facilitate pathogens entering this area. In the case of bacterial diseases, respiratory infections are in the first place in terms of frequency: bronchopneumonia, sinusitis, otitis media, mastoiditis, and meningitis [8].

The most common respiratory tract bacteria are *Haemophilus* spp., *Pseudomonas aeruginosa*, and *Streptococcus pneumoniae*. *Haemophilus* spp. occur in the gastrointestinal tract and vaginal mucosa of a healthy person as well [9]. *H. influenzae* is a pleomorphic Gram-negative coccobacillus, a major human pathogen in the genus [10], and it may be responsible for the development of respiratory mucosal infections as well as invasive bacterial infections (e.g., meningitis) [11,12]. Some studies have reported the biofilm-forming properties of *Haemophilus* species, which are largely determined by piluses, proteins, and bacterial DNA stock [13,14,15,16]. Biofilms caused by *H. influenzae* are of particular concern in patients with otitis media and COPD (Chronic Obstructive Pulmonary Disease) since antibiotic treatment results in no cure owing to increased bacterial resistance due to *H. influenzae* biofilms [17,18].

*H. parainfluenzae*, a Gram-negative rod [19] that is a member of the normal oral flora [9] and the urogenital tract [20], has a scale similar to that of *H. influenzae* [21]. Its significance is given primarily by the fact that it is a member of the HACEK group (*Haemophilus*, *Actinobacillus* (more recently *Aggregatibacter*), *Cardiobacterium*, *Eikenella*, *Kingella*), which can cause endocarditis [19,22]. We do not have much data on the biofilm formation of *H. parainfluenzae*, but studies have shown that it is able to form a biofilm in the area of joint surfaces, making treatment difficult [23,24]. In addition, *H. parainfluenzae* biofilm was detected on the surface of the nasal mucosa and pharynx [25].

*P. aeruginosa* is an opportunistic pathogen that is common in either the surrounding nature or the human body but causes disease mostly in the presence of some predisposing factor [26]. It is currently one of the most dangerous multidrug-resistant nosocomial pathogens [21,27]. Mucilages such as, e.g., alginate play a role in biofilm development.

The genus *Streptococcus* includes facultative aerobic and anaerobic, immobile Gram-positive bacteria. It is the most common pathogen of lobular pneumonia [28]. It is also a common cause of mucosal infections such as otitis media, sinusitis, pneumonia, sepsis, and meningitis [29,30]. Studies have shown that the most common recurrence of otitis media in children is the inability to control *S. pneumoniae* adherent to the mucosal epithelium of the auditory canal by antibiotic treatment [31,32]. In addition, biofilm structures have been observed in certain areas of the lungs in *S. pneumoniae* infection [32,33].

The xerophytic plant immortelle (*Helichrysum italicum* (Roth) G. Don) belongs to the *Asteraceae* family. It is native to the dry, stony and sandy parts of the Mediterranean [34]. The immortelle is a valuable herb that has been used in folk medicine due to its bile-promoting, diuretic and expectorant effects. In addition, research is underway to support the antioxidant, antibacterial, antiviral, anti-inflammatory, and anti-proliferative effects of its extracts and EOs [35]. It should be noted that there is high chemical variation in immortelle oils, (1) chemotypes with high amounts of nerol and its esters; (2) chemotypes with major compounds β- and α-selinene and (3) chemotypes with major compound γ-curcumene. The use of immortelle EO can reduce edema, bruising and inflammation and it is also responsible for analgesia owing to neryl acetate content [36,37]. Moreover, the EO of *Helichrysum* sp. is also used for cold, flu, bronchitis, cough, asthma and as a decongestant as well [38,39]. Han et al. (2017) demonstrated the wound-healing effect of *H. italicum* EO in clinical trials because the EO showed significant anti-proliferative activity. The antibacterial effect of immortelle EO is in connection with their oxygen-containing monoterpenes [40].

The immortelle EO has an anti-inflammatory effect, although its antibacterial activity is not proven against respiratory tract pathogens such as *Haemophilus* spp. The aim of our study was to investigate the antibacterial and anti-biofilm effect of immortelle EO against the most common respiratory tract bacteria, such as *Haemophilus influenzae*, *H. parainfluenzae*, *Streptococcus pneumoniae*, and *Pseudomonas aeruginosa*. In order to reveal the effect of immortelle EO on bacterial cells, scanning electron microscopic examination was used, as well. To the best of our knowledge, our research group was the first to demonstrate a comprehensive study regarding the antibacterial and biofilm-inhibiting effect of immortelle EO against respiratory tract pathogens.

## 2. Results

### 2.1. Chemical Composition of Immortelle Essential Oil

In order to investigate the main components of immortelle EO, GC-MS and GC-FID analyses were carried out. The main component of immortelle was neryl acetate (21.2%), however, α-curcumene was also found in a high percentage in the sample (15.9%) (Table 1).

### 2.2. Broth Microdilution Test

The minimum inhibitory concentrations (MIC) of immortelle EO and the positive antibiotic controls were determined by microdilution assay (Table 2). Our results showed that immortelle EO has good antibacterial activity. The most sensitive pathogen was the *Haemophilus* spp., with MIC values of 0.312 mg/mL. The highest MIC value, meaning the lowest activity, was measured against *P. aeruginosa* (0.625 mg/mL). Positive antibiotic controls were more effective than the immortelle essential oil.

### 2.3. Biofilm Inhibition Assay

**Figure 1:**
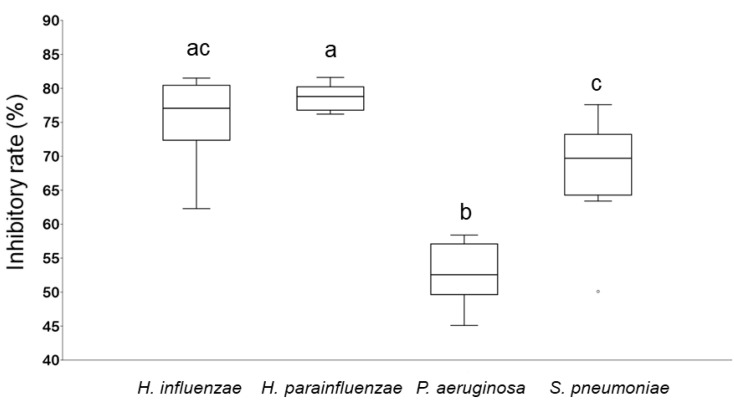
Biofilm degradation activity of immortelle EO against respiratory tract bacteria. The anti-biofilm activity was calculated and demonstrated in terms of inhibitory rate according to the equation: Inhibitory rate = (1 − S/C) × 100% (C and S were defined as the average absorbance of control and sample groups, respectively). Different lowercase letters (a, b, c) above boxes indicate significant differences at *p* ≤ 0.05.

In order to study the biofilm inhibition capacity of immortelle EO, a crystal violet (CV) assay was carried out. The potential of the EO sample to inhibit the biofilm-forming ability of various bacterial strains was examined with MIC/2 concentration. The biofilm inhibition activity was calculated and demonstrated in terms of inhibition rate [42]. The most sensitive bacteria were *H. influenzae* (75.33%) and *H. parainfluenzae* (78.67%) similarly to the antibacterial assay. In contrast, *P. aeruginosa* was the most resistant bacterium, immortelle EO resulted in 52.63% inhibition of biofilm formation. The results clearly showed that immortelle EO was effective against each bacterium, furthermore, *Haemophilus* spp. were more sensitive than *P. aeruginosa* and *S. pneumoniae* (Figure 1).

### 2.4. Membrane Damage Assay

In order to investigate the potential mechanism of action of immortelle EO, the kinetics of bacterial membrane degradation was studied by measuring the degree of bacteriolysis in respiratory tract pathogens. Our results showed that the MIC/4 and MIC/2 concentrations were not effective, but the treatment by MIC and above MIC values resulted in membrane degradation in the case of each bacterium (Table 3) showing the best results with MIC × 2 concentrations. The MIC × 4 value resulted in complete cell lysis. The results of the membrane degradation assay were similar to the antibacterial studies, because *Haemophilus* spp. Were the most sensitive, 96.1% lysis was measured by MIC × 2 concentration of immortelle oil in the case of *H. parainfluenzae*. Furthermore, immortelle EO resulted in 79% lysis against *P. aeruginosa* in the case of MIC × 2 value.

In order to investigate the kinetics of release of the cellular material, time course lysis with MIC × 2 EO solutions was performed. The released cellular material was measured from 10 min to 90 min. The results proved that the lysis was started after 20 min treatment. *P. aeruginosa* was the most resistant: the released cellular material was only 81.2% treated by immortelle EO, after 90 min incubation. The highest membrane degradation was detected at 90 min, against *H. parainfluenzae* (98.6%) (Table 4).

### 2.5. Scanning Electron Microscopy (SEM)

#### Investigation of the Biofilm Structure with SEM

**Figure 2:**
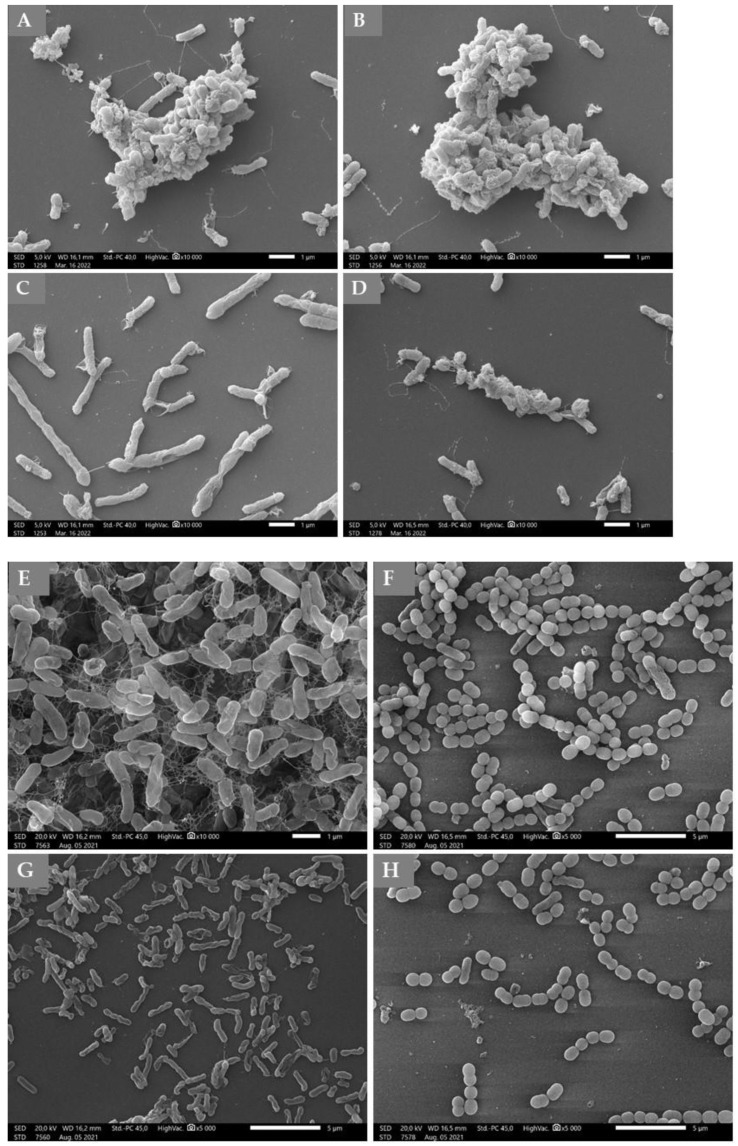
Scanning electron microscopic images of *H. influenzae* (**A**,**C**), *H. parainfluenzae* (**B**,**D**), *P. aeruginosa* (**E**,**G**) and *S. pneumoniae* (**F**,**H**) biofilms. (**A**,**B**,**E**,**F**): Control samples of bacterial strains: (**A**)—*H. influenzae*, (**B**)—*H. parainfluenzae*, (**E**)—*P. aeruginosa*, (**F**)—*S. pneumoniae*. (**C**,**D**,**G**,**H**): Treated bacterial biofilms by Tween40 emulsion of immortelle EO: (**C**)—*H. influenzae*, (**D**)—*H. parainfluenzae*, (**G**)—*P. aeruginosa*, (**H**)—*S. pneumoniae*. Immortelle EO was used in MIC/2 concentration.

The images of the control samples captured the characteristic morphological elements of a mature, three-dimensional biofilm (Figure 2A,B,E,F). The experiments with immortelle EO resulted that the cells attached to the surface, but they did not form biofilm-specific structures (Figure 2C,D,G,H). In the case of Haemophilus influenzae (Figure 2C) it can be observed that the cell division is inhibited because we could detect long and elongated cell shapes.

**Figure 3:**
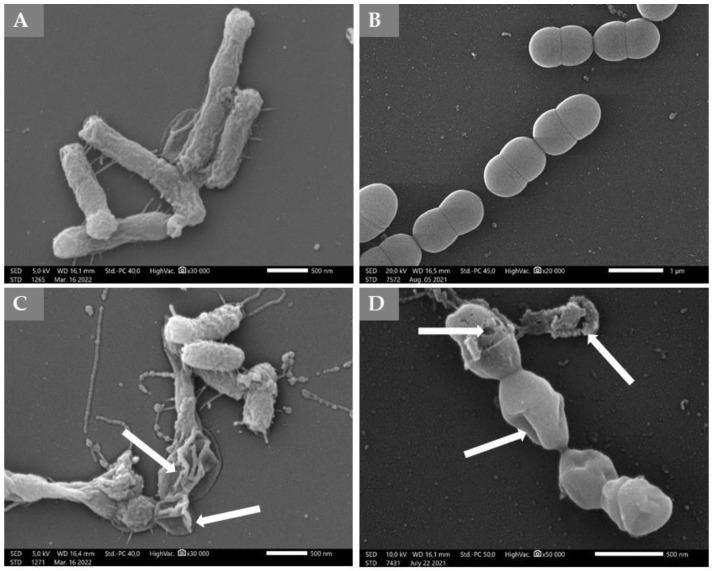
Micrograph—Scanning electron microscopic images of *H. parainfluenzae* and *S. pneumoniae* treated by immortelle EO. (**A**): Untreated control of *H. parainfluenzae*, (**B**): Untreated control of *S. pneumoniae* cells. (**C**): Treated sample of *H. parainfluenzae*, (**D**): Treated sample of *S. pneumoniae*. Immortelle EO was used in MIC × 2 concentration. The arrows show the malformed and burst cells.

In order to demonstrate the membrane damage, SEM images were prepared in the case of the most sensitive Gram-negative (*H. parainfluenzae*) and Gram-positive (*S. pneumoniae*) strains. The electron micrographs obtained from scanning electron microscopy observations showed morphological damage due to the treatment (Figure 3). The micrograph showed that the surface of cells was deformed, which could cause the release of the cellular material. Some cells burst out.

## 3. Discussion

The EOs have become more and more widespread in the prevention of various diseases due to their disinfectant properties and in the alleviation of the symptoms of respiratory, cardiovascular and gastrointestinal diseases. Increasing knowledge of the effects of EOs has shed light on the antimicrobial properties of these natural substances [1,43]. The effect of EOs obtained from aromatic and medicinal plants is highly related to their chemical composition [1]. Studies support that in addition to the main component(s), minor components also have a significant influencing effect [44]. The most widely studied biological activities of EOs are antioxidant capacity as well as antibacterial activity [45,46,47]. EOs can have bacteriostatic effects by inhibiting the growth of bacterial cells—or bactericidal—by killing bacterial cells [48]. To date, phenylpropane derivatives and phenolic components have been shown to have significant antibacterial activity among the constituents of EOs [49,50,51]. In terms of their mechanism of action, their main point of attack is their ability to influence the permeability of the bacterial cell membrane, thus interfering with ion transport (K^+^, Ca^2+^, Na^+^) and inducing protein denaturation processes [52,53] as a result of which enzymatic processes are inhibited [54]. Cell death occurs due to changes in cell membrane permeability, disruption of ion transport, inhibition of mitochondrial processes, and disruption of the intracellular-extracellular ATP balance [47,55]. Overall, the potential controlling mechanism of EOs is mainly due to the action on the multiple stages of biofilm formation. In fact, during the life cycle of biofilms (adhesion, microcolonies formation, and maturation) the anti-biofilm effects are principally related to the inhibition of EPS matrix, the suppression of cell adhesion and the QS system alteration [56].

The antibacterial activity of immortelle EO is well known, even though not against *Haemophilus* spp. The antibacterial effect of immortelle has been studied more thoroughly. Chinou et al. (1996) proved the antibacterial effect of immortelle EO against *S. aureus*, *S. epidermitis*, *E. coli*, *Enterobacter cloacae*, *Klebsiella pneumoniae* and *P. aeruginosa*. Their study showed that staphylococci were sensitive to the treatment, although *P. aeruginosa* and *E. coli* were more resistant to immortelle EO [57]. Tundis et al. (2005) studied the antibacterial activity of *H. italicum* from Calabria and Sardinia. Methanolic extracts from both origins showed the best effect on the Gram-positive bacteria, especially *Micrococcus luteus* [58]. Cui et al. (2015, 2016) evaluated the antimicrobial effect of *H. italicum* EO against food-borne pathogens. The antibacterial activity was evaluated in vitro and on fresh raw vegetables: water spinach, greens and lettuce to determine whether it has the potential to be used for vegetable preservation. Both sets of experiments showed that *H. italicum* inhibits the growth of *E. coli* and *S. aureus* [59,60]. Djihane et al. (2017) proved the antibacterial and antifungal effects of immortelle EO as well. The EO was effective against *Candida albicans*, *Saccharomyces cerevisiae*, *Fusarium* sp., *Aspergillus niger*, *Alternaria alternata* and *Ascochyta rabiei*. The group of Djihane determined the MIC value of immortelle EO agains *S. aureus*, *Micrococcus luteus*, *Enterococcus cereus*, *E. faecalis*, *Bacillus cereus*, *B. subtlis*, *S. epidermidis*, *P. aeruginosa* and *Proteus mirabilis*. However, the study did not prove the antibiofilm effect of immortelle EO [61].

The anti-*Haemophilus* activity of immortelle EO has not been investigated yet. However, our study proved that the immortelle EO had an antibacterial effect, and it could reduce the biofilm formation of *H. influenzae* and *H. parainfluenzae* as well. Our results showed that *H. parainfluenzae* was more sensitive compared to *H. influenzae*.

Furthermore, the antibacterial and anti-biofilm effects of immortelle EO against *S. pneumoniae* have not been investigated yet. However, the antibacterial effect of the ethanolic solution of immortelle was proved against *S. mutans*, because the ethanolic extract inhibited the growth of the bacterium and interfered with the cariogenic effects of *S. mutans* [62], one of the main microorganisms responsible for dental caries [63].

Overall, this study highlighted the effectiveness of immortelle EO against respiratory tract bacteria used in our experiments. We could demonstrate that the immortelle EO treatment damaged the bacterial cell membrane structure and influenced the biofilm formation with the degradation itself.

The novelty of our work is that we proved the antibacterial and biofilm inhibitory effect of immortelle EO against *Haemophilus* spp. And *S. pneumoniae* firstly. Furthermore, our study summarizes and compares the antibacterial and biofilm inhibitory effects of immortelle EO in the case of respiratory tract pathogens.

## 4. Materials and Methods

### 4.1. GC-FID and GC-MS Analysis

The EO sample (batch number: OF44305/160621) was obtained from a Hungarian company (Panarom Kft., Budapest, Hungary) as a commercial oil. Immortelle oil was diluted in ethanol (10 µL/mL) and 1 µL of oil sample was injected (injector temperature 250 °C) in split mode (split ratio 1:50). Agilent 6890N/5973N GC-MSD (Santa Clara, CA, USA) equipped with an Agilent SLB-5MS capillary column (30 m × 250 µm × 0.25 µm) was used. The gradient program was applied, and GC oven temperature was increased at a rate of 8 °C/min from 60 °C (3 min isothermal) to 250 °C (250 °C/1 min final isotherm). The carrier gas, helium, was used at 1.0 mL/min (37 cm/s) in constant flow mode. The mass selective detector (MSD) was equipped with a quadrupole mass analyzer and was operated in electron ionization mode at 70 eV in full scan mode (41–500 amu at 3.2 scan/s). The data were evaluated with MSD ChemStation D.02.00.275 software (Agilent, Santa Clara, CA, USA). The identification of the compounds was carried out based on Kovats index (KI) and the recorded spectra with the data of the NIST 2.0 library. Fisons GC 8000 gas chromatograph (Carlo Erba, Italy) was used for GC-FID equipped with an Rt-β-DEXm capillary column (30 m × 0.25 mm i.d., 0.25 µm film thickness, Restek) and nitrogen (6.8 mL/min flow rate) was the carrier gas. A total of 1 µL of ethanol solution of immortelle oil was injected (injector temperature 210 °C) in splitless mode. The detector temperature was 240 °C.

The oven temperature was increased at a rate of 8 °C/min from 60 °C (3 min isothermal) to 230 °C (230 °C/5 min final isotherm). Identification of peaks was made by retention data compared with data obtained by GC-MS and data of standards (Fluka Analytical and Sigma-Aldrich). The percentage evaluation was carried out by area normalization. Three parallel analyses were made, and RSD percentages were below 4.5%.

### 4.2. Cultivation of Test Bacteria

The antibacterial effects of immortelle EO were screened on *Haemophilus influenzae* DSM 4690, *H. parainfluenzae* DSM 8978, *P. aeruginosa* ATCC 27853, *S. pneumoniae* DSM 20,566 in the laboratory of the Department of Medical Microbiology and Immunology (Medical School, University of Pécs, Hungary). Every bacterial strain was grown in 100 mL Brain Heart Infusion Broth (BHI) (Sigma Aldrich Ltd., St. Louis, MO, USA). In the case of *Haemophilus* spp. to the BHI 1 mL of BHI supplement B (Diagon Kft., Budapest, Hungary) and 15 µg/mL NAD solution (1 mg/mL) was added as well. Each bacterium was incubated in a shaker incubator (C25 Incubator Shaker, New Brunswick Scientific, Edison, NJ, USA) at 37 °C and at a speed of 60 rpm for 12 h [64]. The bacterial suspensions were diluted with clear BHI for the appropriate concentrations in the assays.

### 4.3. Preparing the Stock Solution Containing Immortelle EO

To solve the EO in BHI, Tween40 (Sigma Aldricht Kft., Budapest, Hungary) was used as an emulgent. Then, 1% Tween40 was applied for preparing the stock solution containing the immortelle EO. In every assay, Tween40 was used as a solvent/emulgent control. In our experiments, Tween40 did not show an inhibitory effect as an emulgent control [65].

### 4.4. Minimum Inhibitory Concentration (MIC)

The minimum inhibitory concentrations (MIC) were determined with the broth microdilution method. From each bacterium, 100 μL solution (10^5^ cfu/mL) was measured to the wells of a 96-well microtiter plate. Stock solutions from the EO (3 or 5 mg/mL; 3 mg/mL in case of *S. pneumoniae*, 5 mg/mL in case of *Haemophilus* spp., *P. aeruginosa*) were prepared in BHI using 1% Tween40 as emulgent and serial two-fold dilution was made up to (0.0937 mg/mL in case of *S. pneumoniae*, 0.0781 mg/mL in case of *Haemophilus* spp., *P. aeuruginosa*). From each solution, 100 μL was added to the treated wells. After incubation (24 h, 37 °C) absorbance was measured at 600 nm with a microtiter plate reader (BMG Labtech, SPECTROstar Nano, Budapest, Hungary). The negative control was the clear BHI, the positive control was the untreated bacterial suspension. The average of the six replicates was calculated and then the mean of the negative control was subtracted from the value obtained. Absorbance lower than 10% of the positive control samples, i.e., growth inhibition of 90% or more, was considered as the MIC value [66]. During the assay, antibiotics were used as positive controls. The positive control was gentamicin (Gentamicin Sandoz, solution for injection, 80 mg/2 mL) against *P. aeruginosa*, imipenem (Imipenem/Cilastatin Kabi 500 mg/500 mg powder, solution for infusion; stock solution: 0.4 mg/mL) against *S. pneumoniae*, and amikacin (Likacin 250 mg/mL solution for injection, Lisapharma S.p.A.) against *Haemophilus* spp.

### 4.5. Biofilm Inhibition Assay

The bacterial biofilms were prepared in a 96-well microtiter plate. A total of 200 µL of bacterial culture (10^8^ cfu/mL) was added into each well. After that, the microtiter plate was incubated for 4 h, at 37 °C allowing the cells to adhere on the surface of the wells. Then the non-adherent cells were washed with physiological saline solution. The EO was used in MIC/2 concentrations for the treatments. The EO was diluted in BHI, and 1% Tween40 as emulgent was used to prepare a stock solution. From this solution, 200 µL was added to each well. After the treatments, the microtiter plates were incubated again at 37 °C for 24 h. After washing the non-adherent cells with physiological saline solution, the adherent cells were fixed with methanol (15 min). The biofilms were dyed with 0.1% crystal violet solution for 20 min. The redundant dye was removed with 33 *w*/*w*% of acetic acid. Then the absorbance was measured (595 nm) with a microtiter plate reader (BMG Labtech SPECTROstar Nano, Budapest, Hungary). All tests were carried out in three replications [67].

### 4.6. Membrane Damage Assay

The release of cellular material was examined in respiratory tract pathogens. In the membrane damage assay, not the cell wall is damaged, but the permeability of the membrane increases. The absorbance of the supernatant of 1 mL bacterial suspension containing 10^8^ cfu/mL in PBS (phosphate buffer saline) was measured at 260 nm. The bacterial cells treated with EO were suspended in PBS containing MIC/4, MIC/2, MIC, MIC × 2, and MIC × 4 concentrations of EO for 1 h. Control cells were suspended in PBS without EO treatment. Using the MIC × 4 concentrations of EO, the comprehensive cell lysis was achieved.

In order to study the kinetics of membrane degradation, the bacterial cells suspended in PBS containing MIC × 2 EO was treated for different periods of time: 0, 10, 20, 40, 60, and 90 min. After each treatment, cells were centrifuged (Neofuge 15R, Lab-Ex Ltd., Budapest, Hungary) at 11.107× *g* rpm for 2 min, and the absorbance of the supernatant was determined at 260 nm with Metertech SP-8001 (Abl&e-Jasco Ltd., Budapest, Hungary) spectrophotometer. The results were expressed in the percentage of leaked material at 260 nm compared to the untreated cells [68].

### 4.7. Scanning Electron Microscopy (SEM)

SEM was used to visualize the structural modifications of biofilms and the signs of membrane damage after treatment of EO samples. For biofilm formation, 5 mL of each bacterial culture (10^8^ cfu/mL) was added into a sterilized bottle. Sterile coverslips were placed in the bottle and served as the attaching surface for the cells. The plates were incubated for 4 h at 37 °C, then the planktonic cells and BHI were washed out. For treatment of developing biofilms, 5 mL from MIC/2 EO was added. The untreated coverslips were used as a control. After incubation (24 h, 37 °C), the supernatant was removed, and the bottles were washed with physiological saline. The preparation of the samples for electron microscopy was performed with 2.5% glutaraldehyde for 2 h at room temperature (RT) to fix the biofilms formed on the coverslips. For dehydration of biofilms, different ethanol concentrations (50, 70, 80, 90, 95, 98%) were used at room temperature for 2 × 15 min. Finally, t-butyl-alcohol: absolute ethanol mixed in 1:2, 1:1 and 2:1 ratios were added to the samples (each case for 1 h, RT). Then, the samples were dehydrated with absolute t-butyl alcohol for 2 h (RT). The samples were stored at 4 °C for 1 h and freeze-dried overnight. The sample was coated with a gold membrane and observed with a JEOL JSM IT500-HR scanning electron microscope (Jeol Ltd., Tokio, Japan) [66].

### 4.8. Statistical Analysis

The data were compared with one-way ANOVA with Tukey’s pairwise comparisons. Differences were considered statistically significant at *p* ≤ 0.05. All statistical data were calculated using Past statistic software (Version 4.10) [69].

## 5. Conclusions

This study proves that immortelle EO has antibacterial activity against respiratory tract bacteria, such as *Haemophilus influenzae*, *H. parainfluenzae*, *P. aeruginosa* and *S. pneumoniae*. Therefore, we conclude that immortelle EO could be beneficial for certain respiratory infections, but in order to use them with sufficient safety, further investigations are required.

## Figures and Tables

**Table 1:** Percentage composition of immortelle oil. Values are averages of three parallel measurements.

Compounds	KI	Percentage of Compounds (%)
*α*-Pinene	939	6.7
Cymene	1025	1.1
Limonene	1029	2.2
1,8-cineole	1031	0.7
Linalool	1097	2.2
Pentyl-methyl-butanoate	1060	2.3
Terpinene-4-ol	1177	1.1
α-Terpineol	1189	0.8
Nerol	1230	0.9
Neral	1235	0.5
Neryl acetate	1362	21.2
Cyclosativene	1371	0.7
*α*-Copaene	1377	4.5
*β*-Longipinene	1401	8.8
*α*-*cisz*-Bergamotene	1413	2.1
*β*-Caryophyllene	1419	0.5
α-*trans*-Bergamotene	1435	4.2
Geranyl propionate	1476	4.0
Citronellyl isobutyrate	1482	1.8
*α*-Humulene	1464	0.5
*γ*-Selinene	1484	3.2
*α*-Curcumene	1481	15.9
*β*-Selinene	1490	7.2
α-Selinene	1498	1.7
*γ*-Cadinene	1514	0.2
*δ*-Cadinene	1523	0.2
Caryophyllene oxide	1583	4.2
Globulol	1585	0.7
Sum		100

KI: Kovats Index [41].

**Table 2:** The minimum inhibitory concentrations (MIC, mg/mL in case of essential oil; MIC: µg/mL in case of antibiotics) of antibiotics and immortelle essential oil on respiratory tract bacteria.

	*P. aeruginosa*	*S. pneumoniae*	*H. influenzae*	*H. parainfluenzae*
immortelle EO	0.625	0.375	0.312	0.312
gentamicin	2	-	-	-
imipenem	-	0.4	-	-
amikacin	-	-	0.8	0.8

**Table 3:** The effect of immortelle EO at different concentrations on the release of cellular material, absorbing at 260 nm.

Concentrations	*P. aeruginosa*	*S. pneumoniae*	*H. influenzae*	*H. parainfluenzae*
	A_260_ (%)			
MIC/4	0	0	0	0
MIC/2	0	0	0	0
MIC	32.1 ± 1.2	36.3 ± 1.9	40.8 ± 2.9	46.9 ± 0.8
MIC × 2	79.0 ± 1.5	83.7 ± 2.2	90.2 ± 2.4	96.1 ± 2.1
MIC × 4	100	100	100	100

Data are the release values presented in percentage vs. total ± SD (n = 3). The degree of bacteriolysis was expressed in percentage compared to the MIC × 4 concentration.

**Table 4:** The effect of immortelle EO on the release of cellular material, absorbing at 260 nm, from respiratory tract pathogens.

	*P. aeruginosa*	*S. pneumoniae*	*H. influenzae*	*H. parainfluenzae*
Time (min)	A_260_ (%)			
0	0	0	0	0
10	0	0	0	0
20	48.2 ± 2.8	52.1 ± 1.2	63.2 ± 1.9	69.8 ± 2.0
40	50.2 ± 0.8	55.3 ± 2.5	69.2 ± 2.1	70.7 ± 3.0
60	79.1 ± 1.5	83.7 ± 2.2	90.2 ± 2.4	96.1 ± 2.1
90	81.2 ± 2.8	89.8 ± 3.1	95.9 ± 0.9	98.6 ± 0.7

Data are the release values presented in percentage vs. total ± SD (n = 3).

## Data Availability

Not applicable.

## Sample Availability

Samples of the compounds are available from the authors.
